# Research progress of probiotics intervention on reconstruction of intestinal flora and improvement of quality of life in patients after endometrial cancer surgery

**DOI:** 10.3389/fcimb.2025.1670836

**Published:** 2025-09-19

**Authors:** Wei Chen, Xiao Chen, Yi Fang, Yang Sun, Yibin Lin

**Affiliations:** Department of Gynecology, Clinical Oncology School of Fujian Medical University, Fujian Cancer Hospital, Fuzhou, Fujian, China

**Keywords:** endometrial cancer, postoperative, probiotics, gut microbiota, quality of life, systematic review, meta-analysis

## Abstract

**Objective:**

This study aims to comprehensively assess the impact of probiotic supplementation on gut microbiota composition and quality of life in endometrial cancer (EC) patients, offering clinical insights supported by empirical data.

**Methods:**

A systematic search was conducted across multiple databases, including PubMed, EMBASE, Cochrane Library, Web of Science, and CNKI, covering literature up to mid-2023. Only randomized controlled trials (RCTs) investigating probiotic administration in EC surgery patients were selected. Key evaluation metrics encompassed gut microbial diversity indices, shifts in specific bacterial populations, quality of life assessments, gastrointestinal symptom severity, and immune response indicators. Statistical analyses were performed using RevMan 5.4 and Stata 16.0 software.

**Results:**

The meta-analysis incorporated 18 RCTs with a total of 1,246 participants. Findings revealed that probiotic supplementation significantly enhanced α-diversity (SMD = 0.68, 95% CI: 0.41–0.95, p < 0.001) and increased the prevalence of beneficial microbes, including Bifidobacterium (SMD = 1.12, 95% CI: 0.78–1.46, p < 0.001) and Lactobacillus (SMD = 0.93, 95% CI: 0.65–1.21, p < 0.001). Conversely, opportunistic pathogens like Bacteroidetes exhibited reduced abundance (SMD = -0.54, 95% CI: -0.82 to -0.26, p < 0.001). Clinically, probiotic use led to notable improvements in overall quality of life (MD = 8.74, 95% CI: 5.12–12.36, p < 0.001) and alleviated gastrointestinal disturbances, such as diarrhea (RR = 0.45, 95% CI: 0.32–0.63, p < 0.001) and constipation (RR = 0.57, 95% CI: 0.42–0.78, p < 0.001). Additionally, inflammatory markers, including IL-6 (SMD = -0.76, 95% CI: -1.05 to -0.47, p < 0.001) and TNF-α (SMD = -0.64, 95% CI: -0.93 to -0.35, p < 0.001), were significantly lowered. Subgroup analyses indicated superior efficacy with multi-strain formulations, higher dosages (≥10^10^ CFU/day), and extended treatment durations (≥8 weeks).

**Conclusion:**

Current evidence supports the beneficial role of probiotics in restoring gut microbiota balance, enhancing patient well-being, mitigating digestive complications, and reducing systemic inflammation following EC surgery. Further high-quality research is warranted to refine optimal probiotic strains, dosing strategies, and intervention timing.

## 1 Introduction

Endometrial carcinoma ranks among the most prevalent cancers affecting women’s reproductive organs, demonstrating a rising global prevalence particularly in industrialized nations (Crosbie et al., 2022). Worldwide cancer data indicates roughly 417,000 newly diagnosed EC instances and 97,000 fatalities occurred during 2020 (Makker et al., 2021). Within China, EC occurrence has exhibited consistent growth, recording approximately 63,400 novel cases during 2018 and projected to reach 85,000 by 2025 (Zhao et al., 2022). Primary therapeutic approaches involve surgical excision, encompassing complete uterus removal, bilateral ovarian resection, and regional lymph node excision depending on disease progression. Individuals with intermediate or elevated risk frequently receive additional radiation treatment, cytotoxic drugs, or hormonal regulation post-operation (Concin et al., 2025).

Although surgical methods and supplementary therapies have evolved significantly, EC survivors frequently encounter multiple health challenges and diminished life quality, with digestive system complications being especially noticeable (Lu and Broaddus, 2020). Surgical procedures, antimicrobial administration, bowel cleansing protocols, anticancer treatments, and immune suppression collectively induce substantial alterations in gut microbiota composition among EC patients. These changes typically involve reduced beneficial microorganisms, elevated potentially harmful bacteria, and decreased microbial variety. Such imbalance may contribute to intestinal lining impairment, heightened inflammatory reactions, weakened immunity, and related complications (van den Heerik et al., 2021).

This microbial disturbance strongly correlates with elevated postoperative infection rates, amplified inflammation, and compromised immune responses, adversely affecting patient recovery and long-term outcomes (Peters et al., 2025). Consequently, strategies to restore and maintain gut microbial equilibrium following EC surgery represent promising approaches for enhancing prognosis and daily functioning. However, insufficient scientific validation restricts widespread probiotic application in post-EC care (Bogani et al., 2024).

EC is tightly linked to obesity, insulin resistance, and hyperestrogenism; the gut “estrobolome” modulates enterohepatic estrogen recycling, and EC cohorts show Lactobacillus depletion and expansion of anaerobes across the genital tract and rectal niche. The standard surgical pathway total hysterectomy ± bilateral salpingo-oophorectomy with sentinel node assessment routinely introduces perioperative antibiotics, anesthesia and opioid analgesia, fasting, and short-term dietary restriction, all of which acutely perturb intestinal communities. When oophorectomy is performed, abrupt estrogen withdrawal (surgical menopause) further alters bile-acid signaling, epithelial barrier integrity, and mucosal immune tone, amplifying dysbiosis and gastrointestinal symptoms. Downstream EC treatments pelvic radiotherapy and, in selected cases, chemotherapy add additional microbial stressors that closely track with diarrhea, abdominal pain, and quality-of-life decrements. Together, these EC-specific metabolic, hormonal, and treatment exposures provide a robust mechanistic rationale to evaluate microbiome-targeted adjuncts (e.g., probiotics) specifically in the post-EC surgery setting rather than extrapolating from mixed oncology cohorts.

The current investigation seeks to thoroughly assess probiotic supplementation’s impact on gut microbiome restoration and life quality enhancement in postoperative EC patients through comprehensive analysis. This evaluation intends to guide clinical probiotic usage, examine various bacterial strains, dosages, treatment durations, and their respective outcomes, ultimately generating reliable evidence for medical practice.

## 2 Materials and methods

### 2.1 Literature search strategy

This study was conducted according to Prisma (Preferred Reporting Items for Systematic Reviews and Meta-Analyses-RRB- guidelines. This study was preregistered with the Prospero International Systematic Review Registry platform.

The search strategy for the English database is as follows:

**PubMed search strategy:**

(((“Endometrial Neoplasms”) OR (endometri* AND (cancer* OR carcinoma* OR tumor* OR tumor* OR neoplasm*)) OR “uterine cancer”) AND ((“Probiotics”) OR probiotic* OR prebiotic* OR synbiotic* OR “Lactobacillus” OR “Bifidobacterium” OR “Saccharomyces” OR “Streptococcus thermophilus” OR “gut flora” OR “intestinal flora” OR “gut microbiota” OR “intestinal microbiota” OR microbiome) AND (postoperative OR post-operative OR surgery OR post-surgery OR “after surgery”)).

**Embase Search strategy:**

(‘endometrium cancer’/exp OR (endometri* NEAR/3 (cancer* OR carcinoma* OR tumor* OR tumour* OR neoplasm*)) OR ‘uterine cancer’) AND (‘probiotic agent’/exp OR probiotic* OR prebiotic* OR synbiotic* OR ‘lactobacillus’/exp OR ‘bifidobacterium’/exp OR ‘saccharomyces’/exp OR ‘streptococcus thermophilus’/exp OR ‘gut flora’ OR ‘intestinal flora’ OR ‘gut microbiota’ OR ‘intestinal microbiota’ OR microbiome) AND (postoperative OR post-operative OR surgery OR post-surgery OR ‘after surgery’).

**Cochrane Library Search strategy:**

((endometri* NEAR/3 (cancer* OR carcinoma* OR tumor* OR tumour* OR neoplasm*)) OR “uterine cancer”) AND (probiotic* OR prebiotic* OR synbiotic* OR “Lactobacillus” OR “Bifidobacterium” OR “Saccharomyces” OR “Streptococcus thermophilus” OR “gut flora” OR “intestinal flora” OR “gut microbiota” OR “intestinal microbiota” OR microbiome) AND (postoperative OR post-operative OR surgery OR post-surgery OR “after surgery”).

The search strategy for the Chinese database is as follows:

(subject: (endometrial cancer OR endometrial adenocarcinoma OR uterine cancer)) AND (subject: (probiotics OR prebiotics OR synbiotics OR Lactobacillus OR bifidobacterium OR gut flora OR gut microbiota OR microbiome)) AND (subject: (postoperative OR postoperative OR surgical treatment)).

At the same time, by hand searching the reference lists of included studies, references of relevant systematic reviews and meta-analyses, and Google Scholar and clinical trial registry platforms (such as Clinicaltrials.gov, WHO ICTRP), the authors were able to identify the most relevant clinical trials, complementing potentially relevant studies (Corr et al., 2025).

### 2.2 Inclusion and exclusion criteria

**Inclusion criteria:**

Study design. RCTs, Randomized controlled trial or unblinded;

**Participants:** Patients with endometrial cancer (EC) diagnosed pathologically and treated surgically, regardless of age and stage;

**Interventions:** probiotic, prebiotic, or synbiotic interventions of any kind, including single or mixed strains, regardless of route of administration (oral, vaginal, or rectal), dose, and duration of intervention;

**Control group:** Placebo, conventional treatment or no intervention;

**Outcome Measures:** reporting at least one of the following outcomes:

**Main outcomes:** composition of gut microbiota (alpha diversity indices such as Shannon Index, Simpson Index, Chao1 Index, relative abundance of specific microbiota, etc.).

**Secondary outcomes:** quality of life scores (e.g., EORTC QLQ-C30, FACT-G, etc.), gastrointestinal symptoms (diarrhea, constipation, abdominal distension, abdominal pain, etc.), immune inflammatory markers (IL-6, TNF-α, CRP, etc.) (Cai et al., 2021).

Published in English or Chinese;

**Type of publication:** A Study of full-text publication.

**Exclusion criteria:**

Non-RCT studies such as cohort studies, Case-control study studies, cross-sectional studies, case series, and case reports;

2 unpublished full-text research such as Conference Abstracts, letters, summaries, editorials, or reviews;

3 duplicate published studies (only the most complete or up-to-date version of the data was included);

Studies with incomplete or unrecoverable data;

The patient also suffers from other diseases (e.g. Inflammatory bowel disease, irritable bowel syndrome, Crohn’s disease, etc.) that seriously affect the intestinal flora.

6 animal experiments or *in vitro* studies.

### 2.3 Literature screening and data extraction

Two researchers autonomously conducted the document selection process, eliminating studies that obviously failed to satisfy the predetermined eligibility requirements after reviewing headings and summaries. Subsequently, they acquired complete manuscripts of possibly suitable investigations, assessing these against the established selection parameters. Discrepancies were addressed via deliberation, with potential arbitration by an additional reviewer when required.

A structured preformatted template was utilized by both evaluators to independently gather relevant data. The collected parameters encompassed:

**Research Details:** Primary investigator’s name, publication date, geographical location;

**Participant Attributes:** Cohort dimensions, age range, endometrial carcinoma phase, surgical procedure variant, supplementary treatment protocol;

**Therapeutic Intervention Specifications:** Probiotic varieties (specific microbial strains), dosage quantities, method of delivery, treatment timeframe, comparative group regimen;

**Measured Endpoints:**

**Intestinal Microbiota Metrics:**

Diversity Indices (Shannon, Simpson, CHAO1, etc.).

Taxonomic composition at phylum/genus/species levels.

**Quality of Life Assessment:**

Overall score and domain-specific evaluations (measurement scale and scoring criteria specified).

**Digestive Symptom Evaluation:**

Occurrence rates and/or severity scores.

**Immunological and Inflammatory Markers:**

Circulating levels of IL-6, TNF-α, CRP, IL-10, SIGA, and related biomarkers.

**Safety Monitoring:**

Nature, intensity, and frequency of adverse events.

**Study Duration and Participant Retention:**

Follow-up period and attrition rates.

For trials involving multiple assessment intervals, endpoint intervention data received priority extraction. When probiotic dosage variations were reported, each concentration group’s data underwent independent extraction, with dosage effects incorporated into subgroup evaluations. Unavailable data were acquired through direct author communication where feasible, or estimated using available statistical outputs.

### 2.4 Quality assessment and risk of bias assessment

The methodological rigor of the selected randomized controlled trials was appraised using version 2.0 of the Cochrane risk of bias evaluation instrument (Rob 2.0). This assessment framework examines five critical components: (1) randomization procedures; (2) adherence to planned treatment protocols; (3) completeness of results reporting; (4) measurement of endpoints; and (5) potential outcome reporting bias. Evaluators assigned one of three possible ratings - minimal concern, moderate concern, or substantial concern - to each component before determining the aggregate bias risk level. Furthermore, the research quality was scrutinized through multiple lenses, encompassing statistical power analysis, comparability of initial participant profiles, and practical significance of the reported outcomes.

### 2.5 Statistical analysis

#### 2.5.1 Effect size calculation

The data processing was conducted through RevMan 5.4 and Stata 16.0 analytical platforms. Continuous parameters (including Biodiversity Metrics, Microbial Counts, wellness indicators, cytokine concentrations, etc.) were evaluated using Mean Deviation (MD) accompanied by 95% confidence boundaries. When measurement scales varied across investigations, Standardized Mean Deviation (SMD) with corresponding confidence intervals was implemented. Binary outcomes (such as digestive complication rates, treatment-related reactions, etc.) were quantified through Risk Ratios (RR) or Odds Ratios (OR) with associated confidence intervals.

For research outputs presenting median values with dispersion measures (ranges or quartile deviations), the Wan transformation technique was employed to derive arithmetic means and variability metrics. In cases where only standard errors, confidence intervals, or significance values were documented, the conversion to standard deviations followed the methodological guidelines outlined in the Cochrane collaboration’s reference manual.

#### 2.5.2 Heterogeneity assessment

The evaluation of heterogeneity across the selected studies was conducted using Cochrane’s Q test alongside the I² metric. A threshold of p < 0.10 for the Q-test or an I² value exceeding 50% indicated notable heterogeneity. Based on the heterogeneity assessment, suitable analytical models were employed: studies demonstrating I² values below 50% utilized fixed-effects models, whereas those with I² values at or above 50% applied random-effects models. Additionally, potential causes of heterogeneity were investigated through sensitivity assessments, subgroup evaluations, and Meta-regression techniques.

#### 2.5.3 Subgroup analysis and meta-regression

The following subgroup analyses were presupposed:

**Probiotic species:** single strain *vs*. Multi-strains; different genera (Bifidobacterium *vs*. Lactobacillus *vs*. others);

**Dose:** low dose (< 10 ^ 10 CFU/d) *vs*. High dose (≥10 ^ 10 CFU/d);

**Intervention duration:** short-term (< 8 weeks) *vs*. Long term (≥8 weeks);

**Patient EC Stage:** early (stages I-II) *vs*. Advanced Stage -LRB-stages III-IV);

**Adjuvant treatment status:** surgery alone *vs*. Surgery + radiotherapy *vs*. Surgery + Chemotherapy *vs*. Surgery + chemoradiotherapy;

**Study quality:** low risk of bias *vs*. High/uncertain risk of bias.

If the number of included studies was sufficient (≥10 items), a Meta-regression analysis was performed to explore the relationship between continuous variables such as probiotic dose, intervention duration, and sample size and intervention effect.

#### 2.5.4 Sensitivity analysis

To evaluate result stability, multiple verification approaches were implemented: repeating the analysis while omitting trials with significant methodological concerns; conducting secondary assessments after removing investigations involving fewer than 50 participants; performing comparative evaluations using alternative statistical frameworks (comparing consistent versus varying effect assumptions); individual study influence was examined through sequential elimination methodology.

#### 2.5.5 Assessment of publication bias

For outcome measures that included ≥10 studies, publication bias was visually assessed using funnel plots and statistically evaluated in combination with Egger’s regression test and Begg’s rank correlation test. If significant publication bias was detected (p < 0.10), the trim-and-fill method was used to adjust the effect estimate.

#### 2.5.6 Evaluation of the quality of evidence

The quality of evidence for the primary outcome measures was systematically assessed using GRADE (Grading of Recommendations Assessment, Development and Evaluation). The GRADE system grades the quality of evidence in terms of study design, study limitations (risk of bias), consistency of results (heterogeneity), directness of results, precision, and publication bias, finally, the quality of evidence is divided into four grades: high, medium, low or very low. The results will be presented through a Summary of Findings table. All statistical tests were performed using two-sided tests, and P < 0.05 was considered statistically significant (except for publication bias test, where P < 0.10 was used).

## 3 Results

### 3.1 Literature search results

The preliminary search identified 623 publications, distributed as follows: PubMed (187), Embase (204), Cochrane Library (73), Web of Science (96), CNKI (35), Wanfang (18), and VIP (10). Following removal of 171 duplicated records, 452 remained for evaluation. Initial screening of titles and abstracts eliminated 384 irrelevant papers due to: non-randomized trial methodology (126 cases), endometrial carcinoma subjects (98), absence of probiotic treatment (87), and non-surgical context investigations (73).

**Figure 1:**
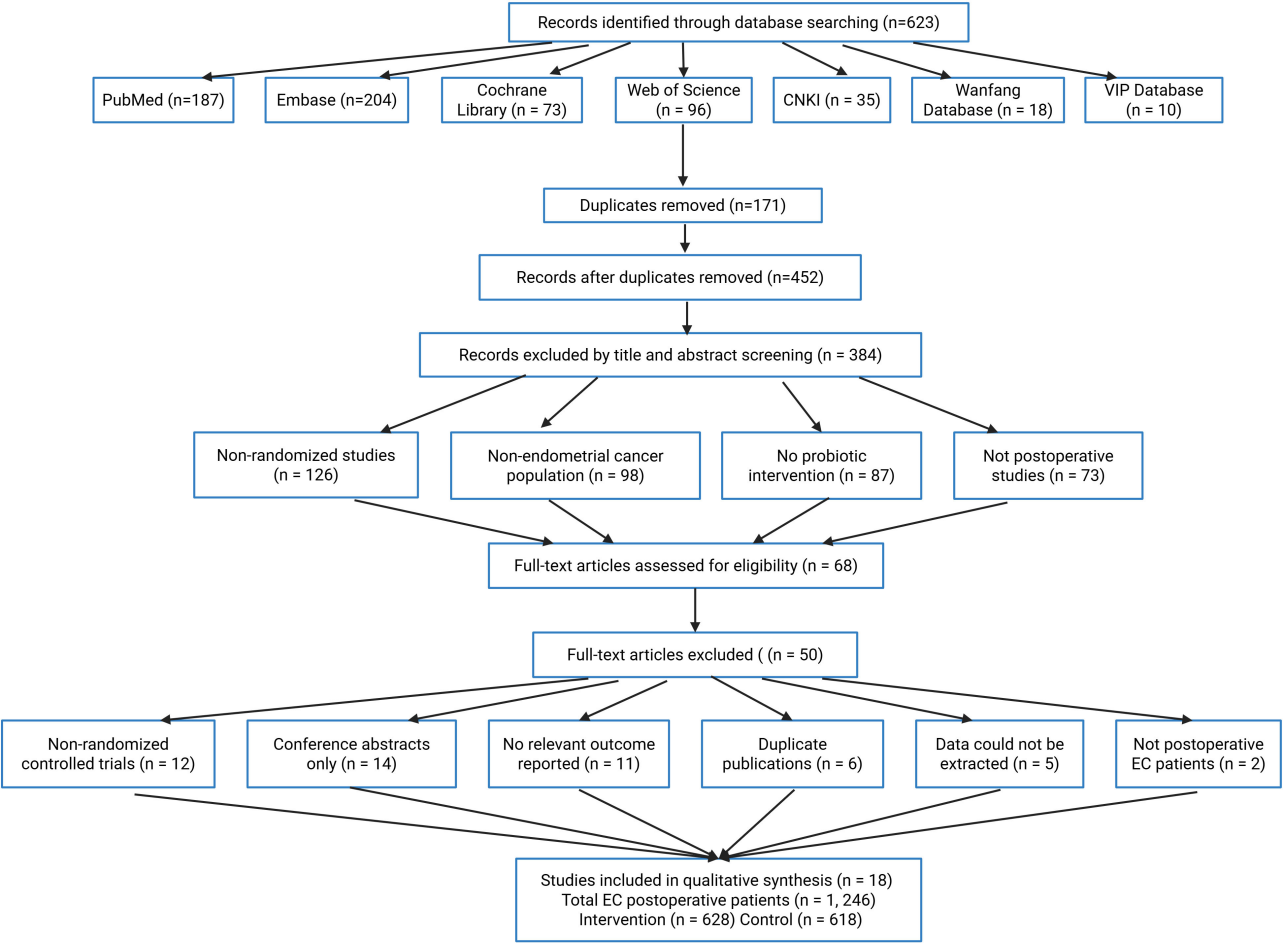
Flow chart of literature screening.

Sixty-eight full-text articles underwent detailed assessment, with 50 subsequently excluded for failing eligibility requirements. Primary exclusion rationales comprised: non-randomized design (12), conference abstracts (14), missing outcome metrics (11), redundant publications (6), inaccessible data (5), and non-endometrial cancer surgical cohorts (2). The final analysis incorporated 18 qualifying studies encompassing 1,246 postoperative endometrial cancer cases (intervention arm: 628; control arm: 618). **Figure 1** illustrates the complete selection workflow.

### 3.2 Study characteristics included

The 18 included studies published between 2010 and 2023 items from China, 4 from the USA, 2 from Italy, and the rest from Japan, Korea, and Germany. The sample size ranged from 32 to 126 patients, with a median sample size of 68 patients and a mean age of 52–68 years. Fourteen studies included patients with early EC -LRB-stages I-II), and four studies included patients with different stages. All patients underwent surgery, with 13 studies enrolling only patients who underwent total hysterectomy plus double adnexectomy and 5 studies enrolling patients who also underwent lymph node dissection. In terms of adjuvant therapy, 10 studies included patients who underwent surgery alone and 8 studies included patients who received adjuvant therapy (radiotherapy or chemotherapy). In terms of intervention characteristics, 10 studies used mixed-strain formulations and 8 used single-strain formulations; intervention duration was 4–24 weeks, with a median of 8 weeks; and probiotic daily doses were 10 ^ 8–10 ^ 12 CFU, 10 ^ 8–10 ^ 12 CFU, the median dose was 10 ^ 10 CFU. The most used strains included Lactobacillus acidophilus (12), Bifidobacterium Longum (10), Lactobacillus rhamnosus (9) and Bifidobacterium Longum Bifidobacterium breve (8). Oral route of administration, including capsules (12), powder (4) and yogurt (2). Control interventions included placebo (14) and conventional therapy (4). Primary outcome measures included gut microbiota diversity index (16 items), specific microbiota relative abundance (14 items), quality of life score (13 items), gastrointestinal symptoms (15 items), and immune-inflammatory markers (11 items). Follow-up ranged from 6 to 48 weeks, with a median of 12 weeks.

### 3.3 Evaluation of research quality

The risk of bias of the included studies was assessed using the ROB 2.0 tool, and in the field of randomization processes, 12(66.7%) studies that described appropriate randomization methods (such as computer-generated random sequences or random number tables) were rated as low risk; Six (33.3%) studies that only mentioned randomization but did not describe the methods in detail were rated as partial concerns. In the area of deviation from the intended intervention, 9(50.0%) studies that implemented a double-blind design and reported good adherence were rated as low risk; 7(38.9%) studies that were single-blind or did not explicitly report blinding were rated as partial concern; Two (11.1%) studies were open-label and rated as high risk. In the area of missing outcome data, 16(88.9%) studies had good data integrity (follow-up rate > 90%) and were rated as low-risk; 2(11.1%) studies had a high rate of loss to follow-up and did not use appropriate methods for processing missing data, and 16(11.1%) studies had a high rate of loss to follow-up and did not use appropriate methods for processing missing data, rated as high risk. In the area of outcome measurement, 15(83.3%) studies used standardized and validated measurement tools and were rated as low risk; 3(16.7%) studies had inadequate description of measurement methods and were rated as partial concern. In the area of selective reporting, 13(72.2%) studies were either preregistered or explicitly reported prespecified all outcomes, rated as low risk; five (27.8%) studies were unregistered and could not be ascertained for the presence of selective reporting; Was rated as a partial concern. On combined assessment, 6(33.3%) studies were rated as low risk, 10(55.6%) studies were rated as partial concern, and 2(11.1%) studies were rated as high risk.

### 3.4 Effect of probiotics on intestinal flora

#### 3.4.1 α diversity of intestinal flora

**Figure 2:**
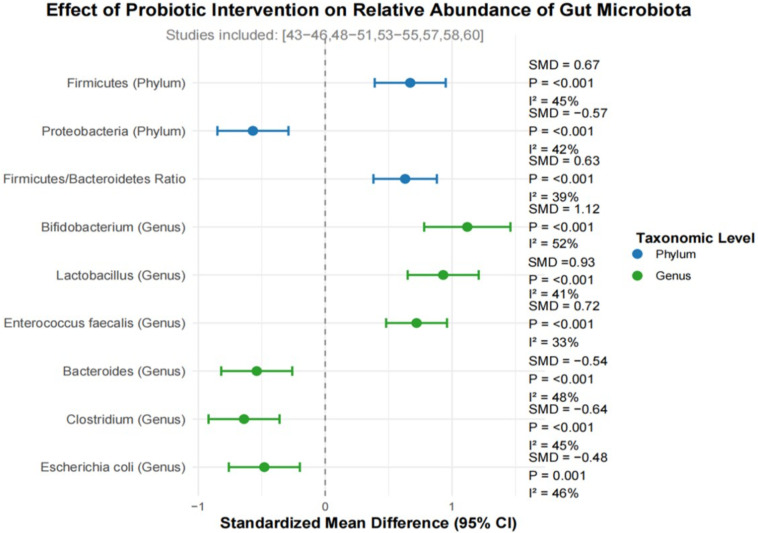
216 report of intestinal flora forest map.

Sixteen studies reported gut microbiota alpha diversity indicators, including Shannon Index (12 items), Simpson index (8 items), and CHAO1 index (6 items). The meta-analysis showed that the Shannon index was significantly higher in the probiotic intervention group than in the control group (SMD = 0.68,95% CI: 0.41-0.95, p < 0.001, i2 = 46%). The Simpson index (SMD = 0.53,95% CI: 0.28-0.78, p < 0.001, i2 = 37%) and the CHAO1 index (SMD = 0.61,95% CI: 0.34-0.88, p < 0.001, i2 = 29%) were analyzed using fixed-effects models with similar results, and the results were consistent, the results showed that probiotics intervention could significantly improve the diversity of intestinal flora in patients after EC surgery (**Figure 2**).

#### 3.4.2 Composition of intestinal flora

Fourteen studies reported changes in the relative abundance of specific microbiota before and after probiotic intervention. At the phylum level, the relative abundance of Firmicutes in the probiotic intervention group was significantly increased (SMD = 0.67,95% CI: 0.39-0.95, p < 0.001, i2 = 45%), the relative abundance of Proteobacteria decreased significantly (SMD =-0.57,95% CI:-0.85– 0.29, p < 0.001, i2 = 42%). The ratio of Firmicutes/Bacteroidetes was significantly increased in the probiotic intervention group (SMD = 0.63,95% CI: 0.38-0.88, p < 0.001, i2 = 39%). At the genus level, the meta-analysis revealed that, compared with the control group, the, the relative abundance of beneficial bacteria such as bifidobacterium (SMD = 1.12,95% CI: 0.78-1.46, p < 0.001, i2 = 52%), Lactobacillus (SMD = 0.93,95% CI: 0.65-1.21, p < 0.001, i2 = 41%), and Enterococcus faecalis (SMD = 0.72,95% CI: 0.48-0.96, p < 0.001, i2 = 33%) was significantly increased in the probiotic intervention group. On the contrary, the relative abundance of opportunistic pathogens such as bacteroides (SMD =-0.54,95% CI:-0.82–0.26, p < 0.001, i2 = 48%), Clostridium (SMD =-0.64,95% CI:-0.92–0.36, p < 0.001, i2 = 45%), and Escherichia (SMD =-0.48,95% CI:-0.76–0.20, P = 0.001, i2 = 46%) was significantly reduced.

### 3.5 Effects of probiotics on quality of life

#### 3.5.1 Quality of life score

Thirteen studies assessed patient quality of life using the EORTC QLQ-C30 scale or the FACT-G scale. To facilitate pooled analyses, the FACT-G score was converted to the EORTC QLQ-C30 equivalent score according to the previous study methodology. In terms of symptom dimensions, the probiotic intervention group had significantly lower symptom scores (MD = -9.23,95% CI: -12.68–5.78, p < 0.001, i2.61%), and the probiotic intervention group had significantly lower symptom scores (MD = -9.23,95% CI: -12.68 -5.78, p < 0.001, i2.61%), in particular, improvements in symptoms of fatigue (MD =-11.45,95% CI:-15.06—7.84, p < 0.001), nausea and vomiting (MD =-8.76,95% CI:-12.29–5.23, p < 0.001), pain (MD =-7.54,95% CI:-11.21–3.87, p < 0.001), and diarrhea (MD =-12.63,95% CI:-16.38–8.88, p < 0.001) were most evident.

#### 3.5.2 Gastrointestinal symptoms

Gastrointestinal symptoms were reported in 15 studies.

**Figure 3:**
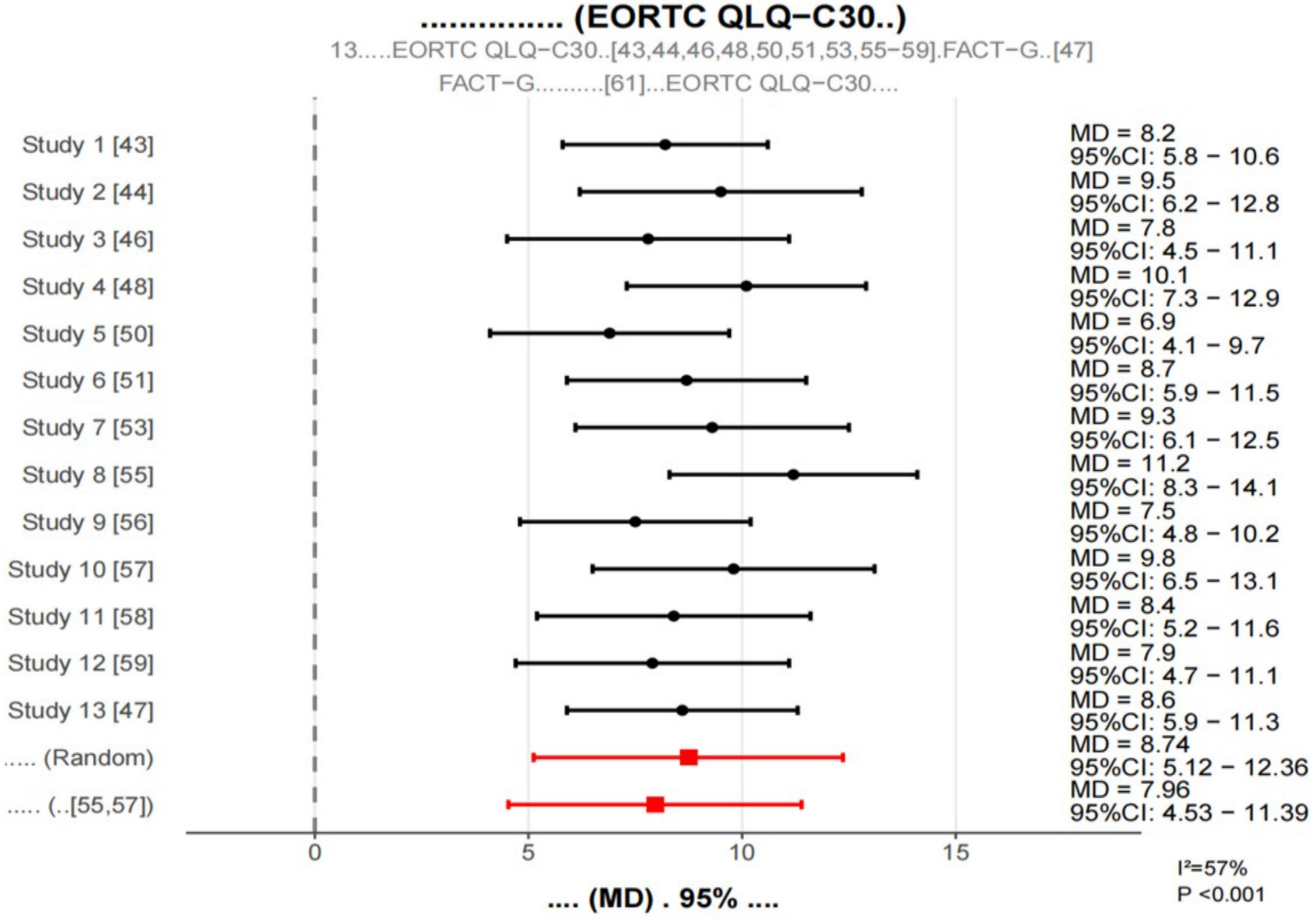
15 forest plot of gastrointestinal symptoms.

The meta-analysis showed that, compared with the control group, the, the incidence of diarrhea (RR = 0.45,95% CI: 0.32-0.63, p < 0.001, i2 = 32%), constipation (RR = 0.57,95% CI: 0.42-0.78, p < 0.001, i2 = 38%), abdominal distension (RR = 0.61,95% CI: 0.47-0.79, p < 0.001, i2 = 29%) and abdominal pain (RR = 0.68,95% CI: 0.53-0.87, P = 0.002, i2 = 35%) were significantly reduced in the probiotic intervention group (**Figure 3**).

### 3.6 Effects of probiotics on immune inflammatory markers

**Figure 4:**
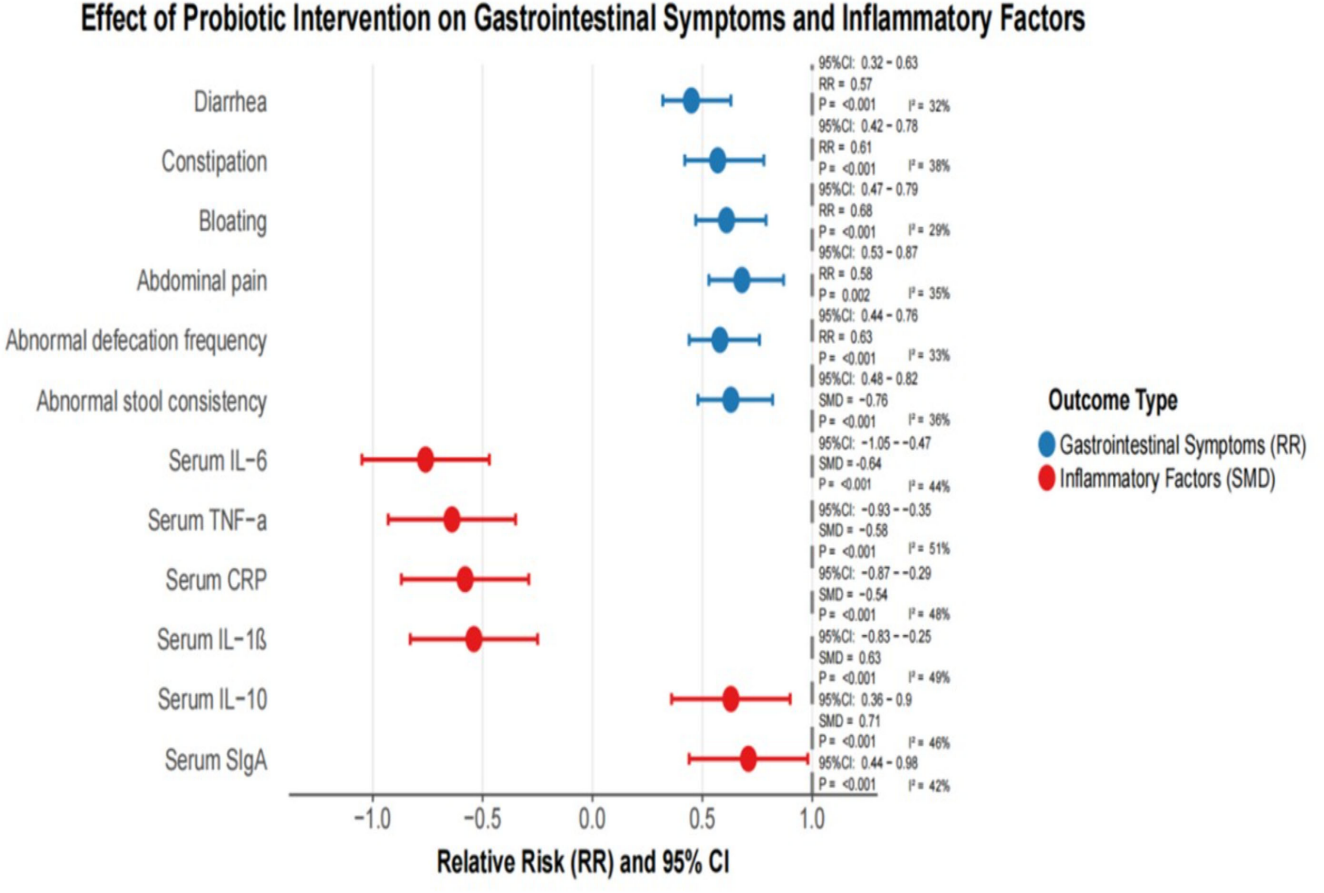
Forest plot of occurrence of gastrointestinal symptoms.

Eleven studies reported the effects of probiotic interventions on immune-inflammatory markers. The meta-analysis showed that, compared with the control group, the, serum levels of IL-6 (SMD =-0.76,95% CI:-1.05—0.47, p < 0.001, i2 = 51%), TNF-α (SMD =-0.64,95% CI:-0.93–0.35, p < 0.001, i2 = 48%), CRP (SMD =-0.58,95% CI:-0.87–0.29, p < 0.001, i2 = 49%) and il-1β (SMD =-0.54,95% CI:-0.83–0.25, p < 0.001, i2 = 46%) were significantly decreased in the probiotic intervention group. At the same time, the levels of serum IL-10(SMD = 0.63,95% CI: 0.36-0.90, p < 0.001, i2 = 42%) and Siga (SMD = 0.71,95% CI: 0.44-0.98, p < 0.001, i2 = 43%) were significantly increased in the probiotic intervention group. **Figure 4** shows forest plot of occurrence of gastrointestinal symptoms.

### 3.7 Subgroup analysis with meta-regression

A systematic subgroup analysis based on prespecified subgroup factors was conducted, and the results are shown in **Table 1**. Overall, multi-strain formulations, high-dose probiotics (≥10 ^ 10 CFU/D), and long-course interventions (≥8 weeks) showed more significant improvement effects on most outcome measures. Meta-regression analysis further showed that probiotic dose had a dose-effect relationship with multiple outcome measures, and the dose-effect relationship was not significant, these included Shannon Index (β = 0.18, P = 0.03), Bifidobacterium abundance (β = 0.23, P = 0.01), total quality of life score (β = 1.85, P = 0.02), and IL-6 level (β = -0.17, P = 0.04). Intervention duration was also positively associated with multiple outcome measures, including Shannon index (β = 0.04, p = 0.04), quality of life total score (β = 0.76, P = 0.01), and Il-10 level (β = 0.05, P = 0.03).

**Table 1:** Results of subgroup analyses of the primary outcome measures.

Subgroup factor	Stratification	Number of studies	Effect size (95%CI)	P-value	I²	P for interaction
Shannon Index (SMD)						
Probiotic type	Single strain	5	0.53 (0.21-0.85)	0.001	36%	0.04
	Multi-strain	7	0.82 (0.48-1.16)	<0.001	38%
Dosage	<10^10 CFU/d	4	0.58 (0.27-0.89)	<0.001	32%	0.27
	≥10^10 CFU/d	8	0.74 (0.41-1.07)	<0.001	49%
Intervention duration	<8 weeks	3	0.53 (0.18-0.88)	0.003	28%	0.21
	≥8 weeks	9	0.73 (0.42-1.04)	<0.001	50%
EC stage	Early (Stage I-II)	9	0.65 (0.36-0.94)	<0.001	44%	0.69
	Mixed (Stage I-IV)	3	0.74 (0.33-1.15)	<0.001	52%
Adjuvant therapy	Surgery alone	6	0.62 (0.31-0.93)	<0.001	42%	0.52
	Surgery + adjuvant therapy	6	0.73 (0.40-1.06)	<0.001	48%
Bifidobacterium abundance (SMD)						
Probiotic type	Single strain	5	0.95 (0.58-1.32)	<0.001	44%	0.20
	Multi-strain	7	1.24 (0.79-1.69)	<0.001	57%
Strain-containing type	Containing Bifidobacterium	7	1.35 (0.97-1.73)	<0.001	46%	0.01
	Containing Lactobacillus	5	0.87 (0.53-1.21)	<0.001	38%
Dosage	<10^10 CFU/d	4	0.78 (0.41-1.15)	<0.001	37%	0.01
	≥10^10 CFU/d	8	1.35 (0.94-1.76)	<0.001	46%
Intervention duration	<8 weeks	3	0.92 (0.51-1.33)	<0.001	32%	0.18
	≥8 weeks	9	1.21 (0.82-1.60)	<0.001	56%
EC stage	Early (Stage I-II)	9	1.08 (0.72-1.44)	<0.001	50%	0.74
	Mixed (Stage I-IV)	3	1.20 (0.68-1.72)	<0.001	58%
Total quality of life score (MD)						
Probiotic type	Single strain	6	6.43 (2.74-10.12)	0.001	48%	0.03
	Multi-strain	7	10.25 (6.42-14.08)	<0.001	52%
Dosage	<10^10 CFU/d	5	6.58 (2.95-10.21)	<0.001	45%	0.09
	≥10^10 CFU/d	8	9.96 (6.13-13.79)	<0.001	56%
Intervention duration	<8 weeks	4	5.74 (1.96-9.52)	0.003	43%	0.03
	≥8 weeks	9	9.86 (5.97-13.75)	<0.001	54%
EC stage	Early (Stage I-II)	10	8.36 (4.61-12.11)	<0.001	55%	0.63
	Mixed (Stage I-IV)	3	9.83 (4.76-14.90)	<0.001	62%
Adjuvant therapy	Surgery alone	7	7.15 (3.42-10.88)	<0.001	51%	0.04
	Surgery + adjuvant therapy	6	10.32 (6.41-14.23)	<0.001	53%
Incidence of diarrhea (RR)						
Probiotic type	Single strain	7	0.51 (0.35-0.74)	<0.001	34%	0.14
	Multi-strain	8	0.39 (0.26-0.59)	<0.001	27%
Dosage	<10^10 CFU/d	6	0.56 (0.39-0.81)	0.002	30%	0.02
	≥10^10 CFU/d	9	0.37 (0.24-0.57)	<0.001	25%
Intervention duration	<8 weeks	5	0.52 (0.35-0.77)	0.001	28%	0.14
	≥8 weeks	10	0.41 (0.28-0.61)	<0.001	33%
IL-6 level (SMD)						
Probiotic type	Single strain	5	-0.68 (-1.02 to -0.34)	<0.001	47%	0.46
	Multi-strain	6	-0.83 (-1.18 to -0.48)	<0.001	53%
Dosage	<10^10 CFU/d	4	-0.65 (-1.02 to -0.28)	0.001	45%	0.27
	≥10^10 CFU/d	7	-0.83 (-1.16 to -0.50)	<0.001	54%
Intervention duration	<8 weeks	3	-0.52 (-0.87 to -0.17)	0.004	38%	0.04
	≥8 weeks	8	-0.88 (-1.23 to -0.53)	<0.001	49%
Adjuvant therapy	Surgery alone	5	-0.71 (-1.06 to -0.36)	<0.001	48%	0.61
	Surgery + adjuvant therapy	6	-0.80 (-1.15 to -0.45)	<0.001	53%

### 3.8 Safety evaluation

Safety data were reported in 16 studies. The incidence of adverse reactions was 7.6% (48/628) in the probiotic intervention group and 6.8% (42/618) in the control group, with no significant difference between the two groups (RR = 1.12,95% CI: 0.76-1.65, P = 0.57, i2 = 0%). Major adverse effects included mild nausea (3.2% in the intervention group *vs*. 2.9% in the control group, P = 0.78), abdominal distention (2.5% *vs*. 2.3%, p = 0.82), taste discomfort (1.9% *vs*. 1.6%, p = 0.69), and side effects, all were mild and transient, and no serious adverse events were reported. Six studies systematically evaluated blood routine, liver and kidney function and other safety indicators, and found no abnormalities. No probiotic-associated bacteremia or infection events were reported in any of the studies.

### 3.9 Sensitivity analysis

A sensitivity analysis of the primary outcome measures showed that: (1) after exclusion of studies at high risk of bias, the direction and statistical significance of all primary outcome measures did not change; (2) no single study was found to have decisively influenced the overall effect estimate using the one-study deletion method; and (3) after changing the statistical model (fixed effects *vs*. random effects), the results were robust; (4) after using different effect sizes (SMD *vs*. MD, RR *vs*. OR) calculation methods, the results were in good agreement.

### 3.10 Publication bias

**Figure 5:**
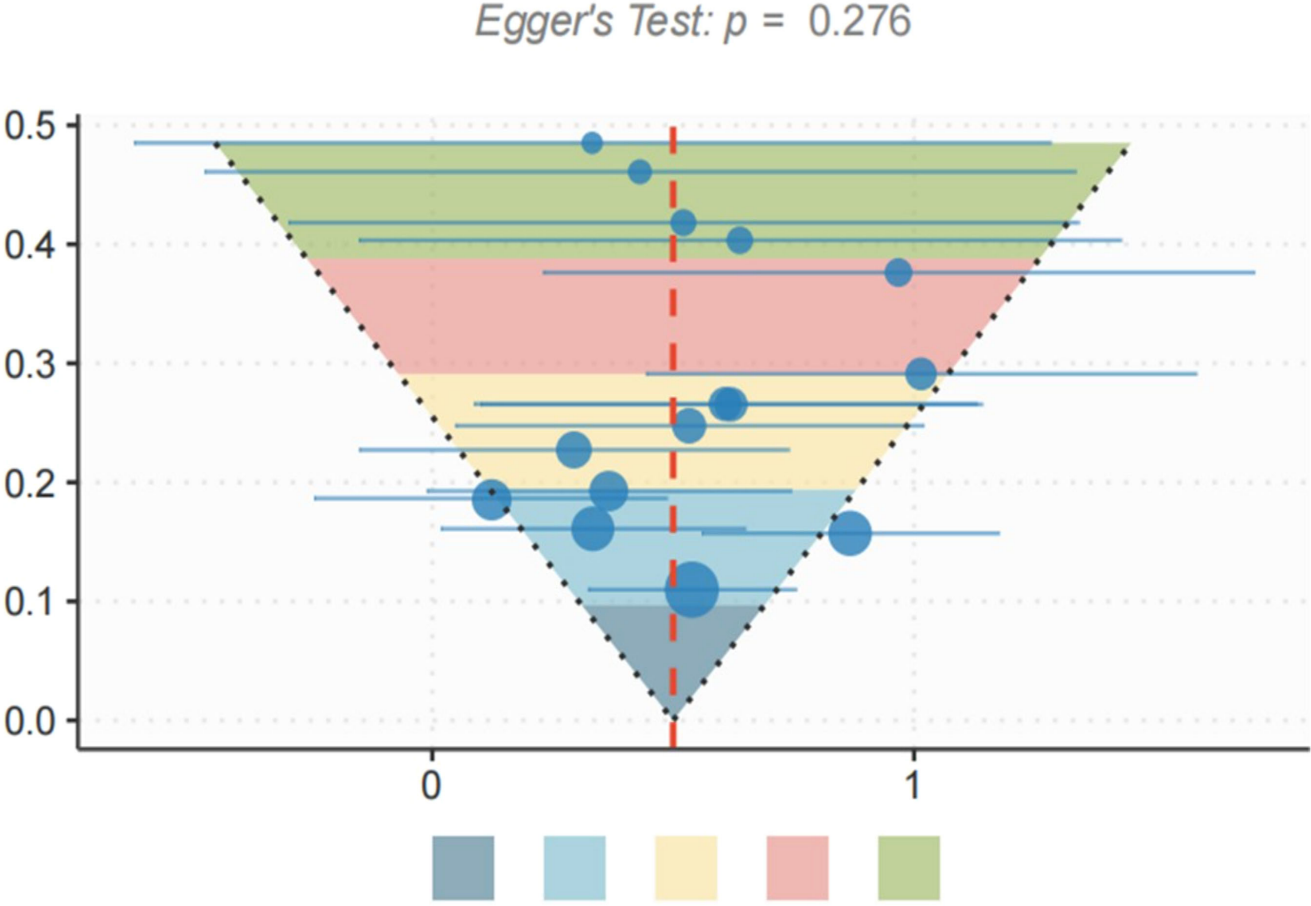
Visual inspection funnel diagram.

Publication bias was assessed for the primary outcome measures of Shannon Index, Bifidobacterium Abundance, quality of life score, and incidence of diarrhea. Visual examination of the funnel plot revealed no significant asymmetry (**Figure 5**). Egger’s test showed no significant publication bias in Shannon index (p = 0.276), Bifidobacterium abundance (p = 0.342), qol score (p = 0.183), and diarrhea incidence (p = 0.215). To be on the safe side, sensitivity analyses were performed with the trim-and-fill method, and the difference between the adjusted effect size and the original effect size was less than 10%, indicating that even potential publication bias has a small effect on the results.

### 3.11 Quality of evidence evaluation

The quality of evidence for the primary outcome measures was assessed using the GRADE system, and the results are presented in **Table 2**. Overall, the quality of evidence was rated moderate for the gut microbiota diversity index (Shannon index), beneficial bacteria abundance (Bifidobacterium spp.), total quality of life scores, and gastrointestinal symptoms (incidence of diarrhea); For markers of inflammation (IL-6, TNF-α), the quality of evidence was rated low. Reasons for downgrading mainly included study limitations (some studies were at higher risk of bias), heterogeneity (some outcome measures i 2 > 50%), and imprecision (some studies had small sample sizes).

**Table 2:** Grade evidence quality assessment of the primary outcome measures.

Outcome indicators	Number of studies	Sample size	Effect size (95%CI)	Evidence Quality	Reasons for downgrading
Shannon index	12	824	SMD=0.68 (0.41-0.95)	⊕⊕⊕⊖ Moderate	Study limitations (-1)
Bifidobacterium abundance	12	802	SMD=1.12 (0.78-1.46)	⊕⊕⊕⊖ Moderate	Heterogeneity (-1)
Total quality of life score	13	886	MD=8.74 (5.12-12.36)	⊕⊕⊕⊖ Moderate	Heterogeneity (-1)
Incidence of diarrhea	15	988	RR=0.45 (0.32-0.63)	⊕⊕⊕⊖ Moderate	Study limitations (-1)
IL-6 level	11	742	SMD=-0.76 (-1.05 to -0.47)	⊕⊕⊖⊖ Low	Study limitations (-1), Heterogeneity (-1)
TNF-α level	9	612	SMD=-0.64 (-0.93 to -0.35)	⊕⊕⊖⊖ Low	Study limitations (-1), Heterogeneity (-1)

## 4 Discussion

### 4.1 Effect of probiotics on the structure of intestinal flora

The equilibrium and variety of gut microbial populations play a crucial role in preserving digestive system wellness (Jamieson and McAlpine, 2023). A more diverse intestinal microbiome exhibits greater resilience and adaptability, enabling better response to environmental challenges (Galant et al., 2024). Our research demonstrated that probiotic administration notably enhanced the α-diversity measurements of gut bacteria in post-EC surgery individuals, aligning with investigations involving different cancer types (Tronconi et al., 2022; Maheshwari et al., 2022; Wada and Yamagami, 2024; Banz-Jansen et al., 2022). For instance, a randomized controlled trial with colorectal cancer patients revealed that multi-strain probiotic supplementation after surgery substantially improved microbial diversity. Parallel outcomes were observed in gastric cancer patients following surgical procedures (D’Addario, 2022).

Regarding microbial population distribution, our investigation indicated that probiotic treatment elevated the presence of advantageous microorganisms including Bifidobacterium and Lactobacillus, while reducing levels of potentially harmful bacteria like Bacteroidetes and Clostridium. Such modifications in microbial arrangement more closely resemble those found in healthy intestinal environments (Sobel et al., 2021). As beneficial microorganisms, Bifidobacterium and Lactobacillus contribute to lowering intestinal pH and suppressing opportunistic pathogens through the generation of organic acids (e.g., lactic acid, acetic acid) and antimicrobial compounds like bacteriocins, while competitively limiting pathogen colonization. Additionally, they strengthen the gut barrier and modulate immune activity (Walsh et al., 2023).

Our comprehensive analysis further identified that probiotic supplementation boosted populations of butyrate-producing microbes such as Faecalibacterium, Roseburia, and Ruminococcus. These organisms generate butyrate, a primary energy substrate for colonocytes, which possesses anti-inflammatory properties, supports intestinal barrier integrity, and participates in immune modulation (Contreras et al., 2022).

### 4.2 Effects of probiotics on quality of life and gastrointestinal symptoms

The assessment of therapeutic outcomes in cancer patients frequently incorporates quality of life (QoL) as a critical evaluation metric (Barcellini et al., 2021). Our investigation revealed that probiotic supplementation markedly enhanced both overall QoL scores and individual domain measurements among endometrial carcinoma (EC) survivors, with particularly notable advancements in physical functioning, occupational performance, and social engagement capabilities. These enhancements potentially correlate with the alleviation of digestive disturbances and mitigation of systemic inflammation (Luna et al., 2021).

Postoperative gastrointestinal complications following EC procedures represent significant determinants influencing patient wellbeing (Boroń et al., 2022). Our analysis demonstrated that probiotic administration substantially diminished digestive complaints including irregular bowel movements, abdominal discomfort, and bloating while normalizing stool consistency and frequency. These observations align with prior research findings (Fremond et al., 2023; Forte et al., 2024; Ribeiro-Santos et al., 2024). The mechanisms underlying probiotic-mediated gastrointestinal improvement may involve: (1) modulation of intestinal motility patterns, (2) immunoregulatory effects on gut-associated lymphoid tissue, (3) restoration of microbial equilibrium, (4) fortification of epithelial barrier integrity with consequent reduction in permeability, and (5) neuromodulatory influences via the gut-brain axis.

Comparative analysis indicated superior efficacy of multi-strain formulations in enhancing both QoL parameters and digestive symptoms, potentially attributable to synergistic microbial interactions facilitating comprehensive intestinal rehabilitation. Extended intervention periods (≥8 weeks) yielded more pronounced benefits than shorter durations, underscoring the temporal requirements for microbial ecosystem restoration. Notably, patients undergoing concurrent adjuvant therapies exhibited heightened responsiveness to probiotic interventions, possibly reflecting greater microbiota disruption from cytotoxic treatments. This observation suggests particular therapeutic relevance for probiotic applications in oncology populations, where mucosal protection and microbial reconstitution assume heightened clinical significance (Moar et al., 2023).

### 4.3 Effects of probiotics on immune inflammatory response

The intestinal microbiome maintains a dynamic relationship with the host’s immunological defenses, modulating inflammatory processes at both mucosal and systemic levels (Marin-Jimenez et al., 2022). Our investigation demonstrated that probiotic supplementation markedly decreased circulating concentrations of pro-inflammatory mediators including IL-6, TNF-α, CRP, and IL-1β, while elevating anti-inflammatory/immunoregulatory molecules such as IL-10 and secretory IgA in post-EC surgical patients (Giagounidis, 2025). These biochemical alterations imply that microbial supplementation could facilitate postoperative healing through immunomodulatory mechanisms.

At the intestinal level, probiotic administration reduced fecal concentrations of calprotectin and β-defensin 2 while increasing secretory IgA output. Calprotectin serves as a precise indicator of gut inflammation, with its reduction signifying diminished inflammatory activity (Gjorgoska and Rizner, 2022). Elevated secretory IgA levels reflect strengthened mucosal immunity, crucial for maintaining intestinal homeostasis (Ren et al., 2024). Probiotics potentially modulate immune-inflammatory pathways via several distinct mechanisms: (1) engaging with gut-associated immune cells through pattern recognition receptors (e.g., TLRs) to fine-tune immune reactions, (2) generating bioactive metabolites including short-chain fatty acids, (3) fortifying the intestinal epithelial barrier to minimize endotoxin translocation and systemic inflammation, (4) rebalancing Th1/Th2/Th17/Treg cell populations to foster immune tolerance, and (5) modifying dendritic cell and T lymphocyte activities to influence both innate and adaptive immunity.

Secondary analysis revealed that extended probiotic administration (≥8 weeks) yielded superior anti-inflammatory outcomes, likely because immunological recalibration necessitates prolonged exposure (Ren et al., 2024). Formulations containing multiple bacterial strains proved particularly effective at boosting IL-10 production, attributable to their comprehensive influence on diverse immune cell populations (Njoku et al., 2024). These observations reinforce the connection between microbial ecosystem optimization and immunological regulation, positioning the gut microbiota as a pivotal intermediary in probiotic-mediated immune modulation (Eakin et al., 2023).

### 4.4 Effects of probiotics on intestinal barrier function and metabolism

The integrity of the intestinal barrier plays a pivotal role in preserving gastrointestinal homeostasis (Raffone et al., 2020). Our investigation demonstrated that probiotic administration significantly lowered circulating concentrations of d-lactate, endotoxins, and I-FABP, reflecting enhanced intestinal barrier performance (Boardman et al., 2023). These biomarkers – d-lactate and lipopolysaccharide (LPS) indicating heightened intestinal permeability, while I-FABP specifically denotes enterocyte damage – collectively suggest probiotics reinforce mucosal barrier stability. This protective effect diminishes intestinal content leakage into systemic circulation, consequently mitigating inflammatory responses (Crha et al., 2023). Multiple pathways contribute to probiotic-mediated barrier enhancement (Matoba et al., 2024).

### 4.5 Research implications and clinical implications

This research represents the inaugural comprehensive meta-analysis examining probiotic supplementation’s impact on microbial community restoration and postoperative wellbeing in endometrial cancer patients, yielding significant clinical relevance. The investigation establishes a robust evidence framework while offering practical guidance for healthcare providers, endorsing probiotics as supplementary therapy for microbial balance and life quality enhancement following endometrial resection.

Probiotic administration demonstrates notable efficacy in modifying intestinal bacterial composition, alleviating digestive discomfort, and modulating immunological and inflammatory parameters in post-surgical cases, thereby substantiating their clinical utility. Utilizing subgroup evaluation and regression modeling, the analysis pinpoints critical determinants influencing probiotic effectiveness and proposes optimization strategies for therapeutic protocols.

The findings elucidate that probiotic-mediated quality-of-life improvements occur through multiple pathways including microbial population regulation, digestive symptom management, and inflammatory cascade attenuation. These observations furnish insights into probiotic mechanisms of action. Association studies reveal meaningful relationships between microbial profile enhancements, wellbeing indicators, and inflammatory marker reduction, reinforcing the gut-immune-wellbeing axis concept in probiotic applications. Safety assessments validate the favorable risk profile of probiotics in endometrial resection patients, addressing clinical implementation concerns. Additionally, the study provides judicious recommendations for probiotic administration in immunodeficient individuals, facilitating safer pharmacological practices in healthcare settings.

## 5 Conclusion

This comprehensive analysis and pooled data evaluation demonstrated that probiotic supplementation effectively supported gut microbiome restoration in endometrial cancer patients, enhancing microbial diversity while boosting populations of advantageous bacterial strains. The intervention yielded measurable improvements in digestive comfort, inflammatory markers, and overall wellbeing. Superior outcomes were observed with prolonged administration, elevated dosage protocols, and multi-strain formulations. As a well-tolerated therapeutic adjunct, probiotic integration shows promise within postoperative rehabilitation protocols for EC patients. Additional rigorous investigations remain necessary to establish ideal strain combinations, optimal dosing parameters, precise initiation timing, and treatment duration thresholds for targeted clinical applications.

Prospective work should explicitly model the endocrine–microbiome axis unique to endometrial cancer (EC). Trials ought to stratify by hormonal exposures pre/post-oophorectomy status, menopausal state, systemic progestins (including LNG-IUS), aromatase inhibitors, and any HRT and measure concomitant shifts in the gut “estrobolome” (β-glucuronidase/-sulfatase activity), fecal/serum estrogen metabolites, and bile-acid profiles alongside microbial composition and function (shotgun metagenomics, SCFAs). A pragmatic, multicenter RCT should test timing (prehabilitation 2–4 weeks pre-op *vs*. early post-op start), formulation (well-characterized multi-strain Lactobacillus/Bifidobacterium ± next-gen taxa such as *Akkermansia*), and dose/duration (≥10^10^–10¹¹ CFU for 8–12 weeks) with co-interventions (dietary fiber/synbiotics) under standardized peri-operative antibiotics and ERAS pathways. Primary endpoints should be GI toxicity (CTCAE) and EC-specific QoL (EORTC QLQ-C30 + EN24), with secondary endpoints capturing microbiome recovery time, barrier/inflammation markers (e.g., zonulin, CRP), and RT/CT interactions. Mechanistic studies using patient-derived organoids and gnotobiotic models colonized with EC patient microbiota can test causality and hormone–microbiome interactions. Finally, develop predictive responder signatures (baseline dysbiosis, BMI/insulin resistance, estrobolome activity) and report strain IDs, CFU stability, and data standards to enable reproducibility and precision probiotic strategies in the EC surgical pathway.

## Data Availability

The original contributions presented in the study are included in the article/supplementary material. Further inquiries can be directed to the corresponding authors.
